# The Prognostic Value and Mechanisms of TMEM16A in Human Cancer

**DOI:** 10.3389/fmolb.2021.542156

**Published:** 2021-02-18

**Authors:** Wenjian Chen, Meng Gu, Chaobing Gao, Bangjie Chen, Junfa Yang, Xiaoli Xie, Xinyi Wang, Jun Sun, Jinian Wang

**Affiliations:** ^1^ Anhui Province Children’s Hospital Affiliated to Anhui Medical University, Hefei, China; ^2^ School of Pharmacy, Anhui Key Laboratory of Bioactivity of Natural Products, Anhui Medical University, Hefei, China; ^3^ Institute for Liver Diseases of Anhui Medical University, Anhui Medical University, Hefei, China; ^4^ Department of Otorhinolaryngology, Head and Neck Surgery, The First Affiliated Hospital of AnHui Medical University, Hefei, China; ^5^ First Clinical Medical College of Anhui Medical University, Hefei, China; ^6^ Anhui Medicine Centralized Procurement Service Center, Hefei, China; ^7^ Department of Neurosurgery, The First Affiliated Hospital of Anhui Medical University, Hefei, China

**Keywords:** cancer, cancer therapy, TMEM16A, biomarker, treatment

## Abstract

As a calcium ion-dependent chloride channel transmembrane protein 16A (TMEM16A) locates on the cell membrane. Numerous research results have shown that TMEM16A is abnormally expressed in many cancers. Mechanically, TMEM16A participates in cancer proliferation and migration by affecting the MAPK and CAMK signaling pathways. Additionally, it is well documented that TMEM16A exerts a regulative impact on the hyperplasia of cancer cells by interacting with EGFR in head and neck squamous cell carcinoma (HNSCC), an epithelial growth factor receptor in head and neck squamous cell carcinoma respectively. Meanwhile, as an EGFR activator, TMEM16A is considered as an oncogene or a tumor-promoting factor. More and more experimental data showed that down-regulation of TMEM16A or gene targeted therapy may be an effective treatment for cancer. This review summarized its role in various cancers and research advances related to its clinical application included treatment and diagnosis.

## Background

Cancer has grown into a major public health problem because it has become the leading cause of death in countries around the world. The GLOBOCAN 2018 estimated 18.1 million new cancer cases and 9.6 million cancer deaths in 2018 (Bray et al., 2018). Nearly, one in six deaths worldwide was caused by cancer. Some clinical data showed that the loss of normal polarity and adhesion of cells was the key to tumorigenesis and metastasis (Yan et al., 2018). The growth and spread of cancer cells in the human body, coupled with the lack of effective methods to detect tumor biomarkers or potential tumor treatment targets, has become the largest element leading to high mortality. Therefore, on the basis of understanding the process of tumor metastasis, finding the ability to inhibit the progress of this process has become an important requirement for solving breakthrough problems in this field (Sun et al., 2019).

**FIGURE 1:**
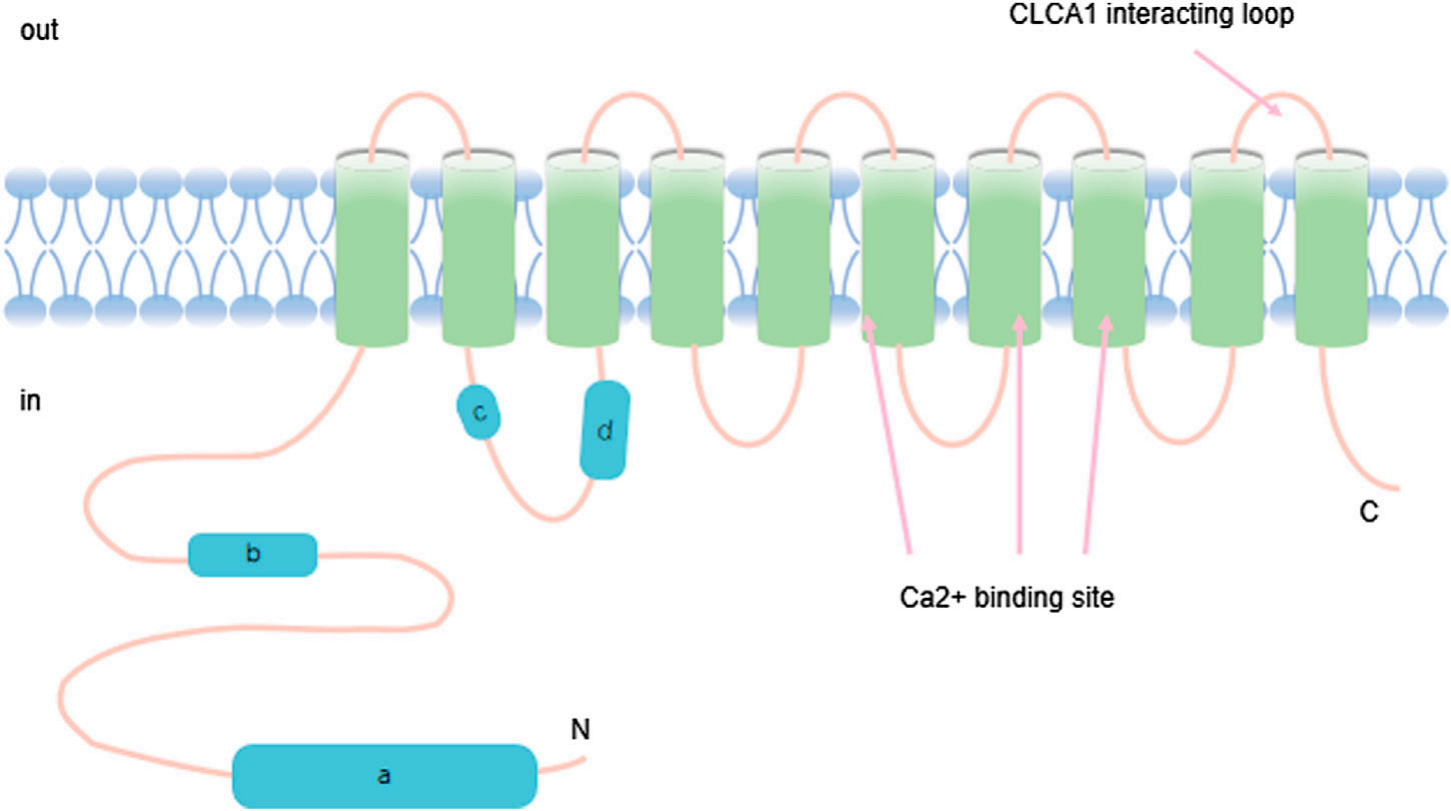
TMEM16A contains ten transmembrane ion domains whose carbon and nitrogen terminals are located on the inner membrane of the cell. The structure also has calcium binding regions.

TMEM16A has eight conserved transmembrane domains as shown in Figure 1. It is a calcium-activated chloride channel (CaCC) named TAOS2, tumor expansion and overexpression-2, DOG1, ANO1 (Figure 1). It is mapped to an area on chromosome 11 (11q13) that is often magnified in cancer cells (Wang et al., 2017). TMEM16A has been detected at a high expression level during the development of a variety of cancers, which is particularly noteworthy (Crottes and Jan, 2019). For example, it was identified as a high-level expressed transcript in gastrointestinal stromal tumors (GIST) (Berglund et al., 2014). TMEM16A profoundly affects the occurrence, proliferation, and migration of a variety of cancers, including breast cancer(BC) (Wang et al., 2019), prostate cancer (PC), head and neck squamous cell carcinoma (HNSCC), lung cancer (LC) (Hu et al., 2019), colorectal cancer, pancreatic ductal adenocarcinoma (PDAC) (Wu et al., 2017; Song et al., 2018; Finegersh et al., 2017; Jia et al., 2015; Sauter et al., 2015). TMEM16A is highly expressed in gastric cancer (GC) and accelerates invasion and migration through transforming growth factor beta (TGF-β) signaling pathway (Liu et al., 2015; Cao et al., 2017). In addition, Jia Linghan et al. have shown that it promoted the growth and invasion of lung cancer (LC) cells. Accumulated evidence showed that knockdown of TMEM16A not only inhibited the migration and invasion of LC cells, but also significantly suppressed the progression and development of LC (Jia et al., 2015). From this perspective, it seems feasible to interfere with tumor growth and metastasis with inhibitors and activators. These data indicated that TMEM16A is involved in the development and progression of cancer. According to the above data, TMEM16A in fact engages in the lifespan of cancer. The review here summarizes the TMEM16A-related studies, which focuses on its function in cancer, and the currently known expression patterns and mechanisms of action.

## Overview of TMEM16A

TMEM16A belongs to the TMEM16 family and has eight transmembrane domains in the membrane including amino and carboxyl terminus (Wang et al., 2017). Of note, it plays a significant role in multiple physiological actions. In addition to its importance in the development and progression of cancer, TMEM16A also plays a role in many normal tissues, including transepithelial secretion (Zhang et al., 2015), smooth muscle contraction (Danielsson et al., 2015; Qu et al., 2019), neural development (Hong et al., 2019), and modulation of neuronal excitability and transduction of sensory-stimulation (Ferrera et al,. 2010). It has been shown that TMEM16A acts as a calcium-activated chloride channel in various tissues, including secretion of epithelial cells, smooth muscle and sensory neurons (Lam and Dutzler, 2018). Considerable evidence showed that TMEM16A regulated airway fluid secretion, intestinal motility, bile secretion etc. (Namkung et al., 2011; Dutta et al., 2016; Kang et al., 2017) In biliary epithelial cells (BECs), Dutta A. K. et al. found that ATP-stimulated TMEM16A activation is through ATP-Ca^2+^-PKCα signaling pathway, and the signaling pathway is an influential regulator of BEC secretion (Dutta et al., 2016). Therefore, TMEM16A may perform a regulatory function in the mechanism of bile formation. Additionally, it is generally accepted that TMEM16A dysfunction is associated with a variety of disease states, including cystic fibrosis, asthma, gastroparesis, hypertension, rotavirus-induced diarrhea and polycystic kidney disease (Zhang et al., 2013; Sondo et al., 2014; Zeng et al., 2018; Ousingsawat et al., 2011; Narayanan et al., 2013; Wang P. et al., 2018). Experimental results have shown an increased expression of TMEM16A in pulmonary artery smooth muscle cells in patients with pulmonary arterial hypertension (PH) (Yamamura et al., 2011; Papp et al., 2019). Thus, TMEM16A may play a crucial role in the development progress of pulmonary hypertension. Interestingly, Tanaka T. et al. found that TMEM16A was expressed diffusely in the cytoplasm of MDCK cysts (Tanaka and Nangaku, 2014).

## The Function of TMEM16A in Various Types of Cancer

**FIGURE 2:**
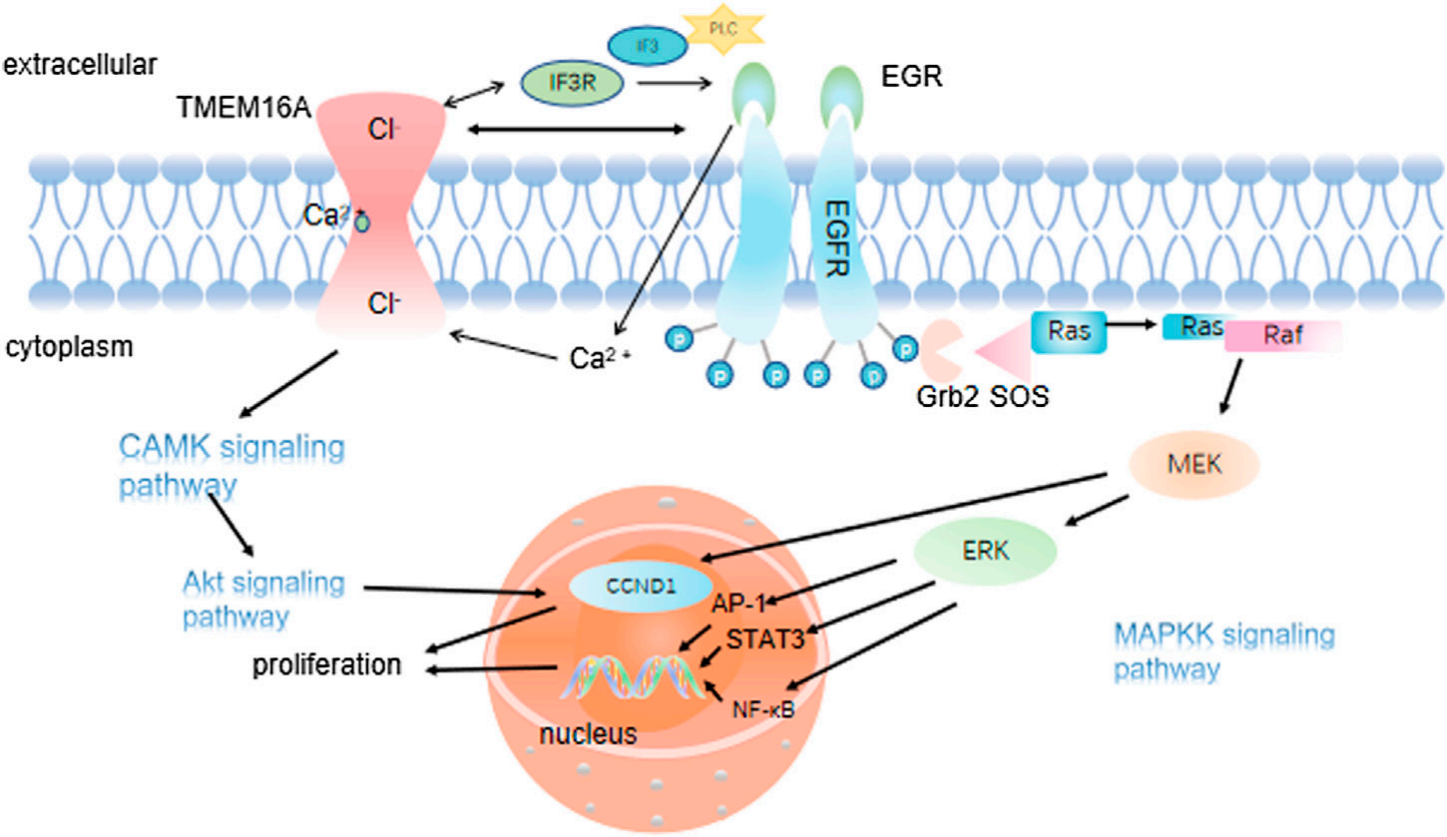
TMEM16A regulates EMT and cancer cell migration and metastasis. 2.1 TMEM16A binds to ezrin-radixin-moesin and regulates the cancer cell EMT; 2.2 TMEM16A activates the intracellular pathway to regulate the expression of Desmosomes, N-cadherin and E-cadherin in the cell, which is essential for cancer cell migration and metastasis; 2.3 By enhancing the expression of RhoA oncogene, TMEM16A causes the dissolution of cell junctions and finally promotes the migration and metastasis of cancer cells.

Increasing evidence suggested that TMEM16A is abnormally expressed in multiple cancers and its dysregulation was associated with cellular functions (Song et al., 2018; Ayoub et al., 2010). What’s more, studies showed that the TMEM16A expression level was significantly inhibited in metastatic HNSCC samples and HNSCC cell lines through MAPK signaling pathway (Hu et al., 2019; Liu et al., 2015; Sun et al., 2014). Simultaneously, TMEM16A could activate EMT and then promote the invasion and migration of cancer cells (Cao et al., 2017) (Figure 2). More importantly, TMEM16A has been demonstrated to be abnormally expressed in a variety of cancers, which could inhibit the invasion and metastasis of cancer cells and improve the prognosis of cancer (Jacobsen et al., 2013; Simon et al., 2013). Dependent on the above evidence, this article takes nine types of cancer as the target, and summarizes the specific role of TMEM16A which can greatly affect cancer activity (Table 1). In addition, its possible clinical value in treatment and prognosis is also discussed.

**TABLE 1:** Effect of TMEM16A on prognosis in various cancers.

Tumor marker	Cancer tape	Expression level	Prognosis	Reference (PMID)
TMEM16A	GC	↑	-	25839162
	EC	↑	-	27016410, 30515811
	CRC	↑	-	26868958
	HNSCC	↑	-	26808319, 26563938, 22912841, 29123240, 22564524
	BC	↑	-	23431153
	GIST	↑	-	27153560

"↑" indicates expression level of TMEM16A is upregulated. "-" indicates expression level of TMEM16A is negatively correlated with the prognostic outcome. The full terms of GC, EC, CRC, HNSCC, BC, GIST are respectively gastric cancer, esophageal cancer, colorectal cancer, head and neck squamous cell carcinoma, breast cancer, gastrointestinal stromal tumor.

### Breast Cancer

BC, one of the most common malignancies in women, is the leading cause of cancer death in women, which accounts for 11.6% of all cancers (Bray et al., 2018). It usually occurs in the mammary gland epithelial tissue of malignant tumors (Sharma et al., 2018). According to statistics, the incidence rate of BC in women aged 40 to 60 is higher than that in other populations (Grybach et al., 2018). As we all know, BC, act as a heterogeneous disease, was divided into different clinical subtypes according to estrogen receptor (ER), progesterone receptor (PR) and human epidermal growth factor receptor 2 (HER2) (Dai et al., 2016). Recently, Wu H. et al. performed immunohistochemistry to investigate the expression level of TMEM16A and Ki67 (a well-known marker for cell proliferation) in 403 BC patients, and investigated whether the expression level of TMEM16A linked to Ki67 in BC subtypes classified by ER, PR, and HER2(Wu et al., 2017). The results suggested that TMEM16A might differentially regulate cell proliferation in a subtype of BC defined by ER, PR, and HER2. TMEM16A promoted cell proliferation in ER-positive, PR-positive, and HER2-negative MCF7 cells, but inhibited cell proliferation in ER-negative, PR-negative, and HER2-negative MDA-MB-435S cells. Additionally, after treatment with tamoxifen, TMEM16A overexpression of PR-positive or HER2-negative BC was associated with a good prognosis. These findings suggested that TMEM16A was used as a potential marker to predict clinical outcome of BC subtypes as defined by the ER, PR and HER2, and Ki67 status (Wu et al., 2017). Wang H et al. found that EGF increased TMEM16A expression via activating the EGFR/STAT signaling pathway. Moreover, TMEM16A silencing effectively reduced BC cell proliferation, especially in combination with EGFR inhibitors (Wang et al., 2019). Meanwhile, the combination could also enhance the response to EGFR/HER family-mediated antibody therapy (Kulkarni et al., 2017), which is of great significance for clinical treatment of BC. Besides, Carneiro A et al. treated HER2-amplified SKBR3 cells with T16A-inhAO1 (a small molecule inhibitor of TMEM16A Cl-channel) and untreated cells served as the control group. Mechanistically, the study confirmed that TMEM16A can affect the occurrence and development of BC by influencing phosphorylation/activation of EGFR and HER2 (Carneiro et al., 2008). Most recently, The Bentires-Alj group found that ANO1 is amplified and highly expressed in BC cell lines and primary tumors (Britschgi et al., 2013). Interestingly, analysis of Oncomine database performed by Duvvuri group indicated that TMEM16A expression was higher in BC tissues compared with that in normal breast tissues (Duvvuri et al., 2012). These studies suggested that although endogenous TMEM16A expression may be high in some normal tissues, malignant cells derived from these tissues further upregulate TMEM16A expression, implicating that TMEM16A may be a potential target gene in malignant transformation.

### Head and Neck Squamous Cell Carcinoma, Oral Squamous Cell Carcinoma and Esophageal Squamous Cell Carcinoma

Worldwide, head and neck cancer accounts for 6% of all cancers, killing more than 300,000 people per year globally (Dharmawardana et al., 2020). Among them, OSCC is the most common type of oral malignancies, accounting for more than 90% (Massano et al., 2006). Since OSCC has the characteristics of predisposition to metastasis and poor prognosis, therefore, researchers are committed to finding effective biomarkers for the detection of OSCC occurrence and metastasis. Huang X. et al. found that TMEM16A was often overexpressed in non-proliferative primary OSCC (Huang et al., 2006). Besides, the TMEM16A expression level was detected in 132/160 OSCC cases/tissues (82.5%), and the protein and mRNA level of TMEM16A was significantly elevated than that of normal tissue (Li et al., 2014). Recently, copper reduction has emerged as a novel therapeutic strategy in the treatment of metastatic cancer (Lopez et al., 2019). ATP7B is a member of the heavy metal transport P-type ATPase, known as the copper transporter, which transfers copper ions out of cells. In a study of ovarian cancer, Kalayda et al. found that ATP7B was mainly located in the trans-Golgi network (TGN) of sensitive cells, while was sequestrated in peripheral compartment of resistant cells. This change in ATP7B cell localization might contribute to sequester platinum drugs in vesicles, thus removing them from their cytotoxic sites. Interestingly, unlike ATP7A, which was mainly involved in intracellular chelation of platinum drugs when exposed to platinum drugs, ATP7B redistributed from TGN to more peripheral vesicles near the cell surface, sequestering platinum drugs into vesicles of the secretory pathway for efflux from cells (Li et al., 2018). Coincidently, Vyas et al. found a strong positive correlation between TMEM16A and ATP7B expression in human HNSCC. TMEM16A overexpression and depletion in HNSCC cell lines caused parallel changes in the ATP7B expression level. Researchers thought that increased oxidative stress in TMEM16A-overexpression cells liberated the chelated copper in the cytoplasm, leading to the transcriptional activation of ATP7B expression, which, in turn, decreased the efficacy of platinum compounds by promoting their vesicular sequestration (Vyas et al., 2019). Sucheta Kulkarni et al. propounded that the interaction of TMEM16A with EGFR and activates EGFR, participating in the proliferation and migration of HNSCC cells (Kulkarni et al., 2017). Furthermore, studies showed that the degree of TMEM16A expression in HNSCC cell was much greater than in normal cells (Bill et al., 2015). More significantly, Duvvuri U et al. found that TMEM16A siRNA leads to the reduction of p-ERK1/2 and cyclin D1. Similarly, the pharmacological inhibition of MEK/ERK and the genetic inactivation of ERK1/2 eliminated the cancer promoting effect of TMEM16A. Based on these results, the expression inhibition of TMEM16A functions to suppress the proliferation, metastasis and invasion in vitro and the growth of tumor in vivo. (Bill et al., 2015). Moreover, TMEM16A regulated the development and transfer of HNSCC cells based on a control over Epithelial-Mesenchymal transition (EMT) by interacting with cytoskeletal Radixin (Shiwarski et al., 2014). Subsequently, Bill A. et al. found that TMEM16A regulates cell migration through a functional complex formed by binding to EGFR. Interestingly, the function of TMEM16A as a chloride channel does not seem to work here (Bill et al., 2015). It was found that the amplification and high expression of TMEM16A in HNSCC cells were more sensitive to gefitinib, suggesting that overexpression of TMEM16A could be used as a predictor of EGFR targeting response in HNSCC therapy. Although EGFR is ubiquitous in HNSCC, only a few patients in clinical trials exercised a response to EGFR-directed therapy (Cassell and Grandis, 2010). Thus, small molecular tyrosine kinase inhibitors (such as gefitinib) and monoclonal antibodies to EGFR (such as cetuximab) as a monotherapy have limited efficacy only in HNSCC patients (Bernier et al., 2009; Cohen et al., 2005; Vermorken et al., 2007). Kulkarni S. et al. proved that the inhibition of TMEM16A and EGFR in a simultaneous manner could improve the reaction to cetuximab. Accumulating evidence suggested that TMEM16A regulated EGFR and HER2 of cellular signaling pathways involved in growth and survival. Furthermore, in the absence of TMEM16A co-targeting, tumor cells may acquired resistance to EGFR/HER inhibitors. In contrast, targeting TMEM16A improved response to biologics designed for EGFR/HER family members (Kulkarni et al., 2017). Furthermore, TMEM16A has a higher expression level in HPV-negative HNSCC. As we all known, the overall survival rate of HPV-positive HNSCC patients is higher than that of HPV-negative HNSCC (Fakhry et al., 2008). By measuring 44 cases of HPV-positive tumors patients and 20 cases of HPV-negative tumors patients, Dixit R. et al. found that the expression level of TMEM16A in HPV-negative HNSCC was significantly higher than that of HPV-positive HNSCC. Further amplification and mRNA expression level of TMEM16A in HPV-negative HNSCC were more severe than HPV-positive by chi-squared analysis and T-test (Dixit et al., 2015). By colony formation experiments, Dixit R. et al. found that HPV-negative HNSCC, rather than HPV-positive HNSCC, was more dependent on TMEM16A. These distinctions are due to differences in the methylation level of the TMEM16A promoter. It can be concluded that the overexpression of TMEM16A is associated with a decreased survival rate of HNSCC patients. Therefore, TMEM16A may be an effective target for clinical treatment of HPV negative HNSCC and OSCC, as well as a biomarker for diagnostic OSCC (Dixit et al., 2015). To assess the effect of TMEM16A on non-11q13 amplified tumors, Kulkarni S. et al. inoculated the nude mice with OSC-19 cells (TMEM16A low expression cells) which was transfected with TMEM16A overexpression plasmid. Meanwhile, the results showed that the number of metastatic tumors was more than that of the control group. This result demonstrated that TMEM16A also contributes to tumor growth and viability even without 11q13 amplification. Additionally, It was found recently that TMEM16A is up-regulated in primary HNSCC, but is down-regulated in lymph node metastasis, but the two processes themselves do not affect each other (Shiwarski et al., 2014). Shiwarski D. J. et al. implanted a specific cell of HNSCC cells expressing endogenous TMEM16A into the tongue of nude mice. Notably, the level of TMEM16A expression in lymph node metastases was reduced. Significantly, TMEM16A knockdown led to a 60 percent increase in the viability of UM-SCC1 cell. In addition, when the UM-SCC1 cell proliferation was inhibited after transduced with TMEM16A lentivral shRNA. By measuring the overexpression of TMEM16A in UM-SCC1 cells, cell migration data can be obtained to show the decrease of migration amount. Moreover, TMEM16A-shRNA tumors formed more than three metastatic nodules in HNSCC nodule models. Taken together, these results suggested that TMEM16A silencing can increase the mobility and migration of HNSCC cells (Shiwarski et al., 2014). However, Huang et al. found that the SCC-25 cell migration rate in TMEM16A silencing group was lower compared with that of the control group. The wound healing time of SCC-25 cells transfected with TMEM16A-shRNA lentivirus was significantly increased, which further enriched our understanding of the role of TMEM16A in OSCC and HNSCC cell migration. Meanwhile, it is investigated that TMEM16A silencing reduced the rate of cell shedding by cell isolation assay. Huang et al. findings indicated that upregulated TMEM16A promotes the proliferation and metastasis of OSCC and HNSCC cells (Huang et al., 2006). In the wound healing experiment, DIDS, anion exchange channel inhibitor, was used to investigate whether calcium-activated chloride channel activity can promote cell migration. The results showed that DIDS could delay the wound healing of control cells, but the decline of TMEM16A expression was not obvious. This indicated that TMEM16A channel activity participates in scc-25 cell migration (Ayoub et al., 2010). In fact, Ruiz C. et al. found that TMEM16A regulates cell volume and causes cell migration in HNSCC BHY cells (Ruiz et al., 2012). Meanwhile, Perez-Cornejo P. et al. found that TMEM16A may play a role in the tissue actin skeleton (Perez-Cornejo et al., 2012). Thus, TMEM16A may accelerate the migration of cancer cells by modulating the controlled changes in cell volume and cytoskeletal tissue. In addition, the high expression of TMEM16A may serve as a biomarker for the generation and metastasis of OSCC, which is crucial for the early detection and treatment of OSCC. In addition, they observed an increased activity of the E-cadherin promoter in T24 cell lines with stable expression of TMEM16A. At the same time, the expression of vimentin protein is reduced. This change contributes to the transformation of mesenchymal cells into epithelial cells and reduces migration (Shiwarski et al., 2014). Of note, the promoter methylation can regulate the expression of TMEM16A in HNSCC cells (Dixit et al., 2015). In summary, it is speculated that altering the TMEM16A promoter methylation in epigenetics may be sufficient to dynamically regulate the morphology and growth characteristics of HNSCC cells during HNSCC progression. Notably, the expression level of TMEM16A in ESCC was significantly increased. ESCC is the main type of esophageal cancer in the world. In the absence of early biomarkers, the five-year survival rate is only about 19% (Jemal et al., 2010). It develops through multiple procedures from dysplasia to invasive carcinoma through carcinoma in situ. Shi Z. Z. et al. found that the mRNA expression level of TMEM16A in ESCC was significantly higher than that in adjacent tissues (Mei et al., 2017). Therefore, Shi Z. Z. et al. speculated that TMEM16A may play a carcinogenic role in the development of esophageal cancer based on moderate dysplasia. Vitro studies have shown that knockdown of TMEM16A inhibited the proliferation of KYSE30 and KYSE510 cells (ESCC cell lines). The results showed that high TMEM16A expression was significantly associated with lymph node metastasis, late tumor staging, and OS severity (Mei et al., 2017). Taken together, the expression of TMEM16A in esophageal carcinoma is expected to provide a new target for molecular diagnosis and gene therapy of ESCC.

### Prostate Cancer

PC originated from glandular epithelial cells and is one of the most common malignancies in males (Wang K. et al., 2018). PC is estimated to be the second leading cause of death in male malignancies (Dijkstra et al., 2014). Besides, it is within the scope of the few neoplasms not well served by 18F-Fluorodeoxyglucose positron emission tomography, namely FDG and PET (Kitajima et al., 2018). Thus, finding effective detection methods is crucial to the treatment of PC. Of note, TMEM16A was overexpressed in PC cells. By examining the histopathological specimens of 17 patients with PC, the high expression of TMEM16A was observed in 76.5% of PC tissues (13/17). In addition, the proliferation of PC-3 cells in TMEM16A silencing group was significantly reduced (Pulukuri et al., 2005). Wen Liu et al. provided convincing evidence that TMEM16A overexpression is associated with the development and progression of metastatic PC (Liu et al., 2012). In their experiments, researchers selected three classic human PC lines for detection, LNCaP, PC-3 and DU145, in which PC-3 cells had high metastatic potential and proliferative capacity (Sanders et al., 2012). Notably, the expression of TMEM16A was increased in LNCaP and PC-3 cells. Silencing of TMEM16A reduced the migration of PC cells. Moreover, the higher Gleason score was associated with higher TMEM16A expression. Therefore, TMEM16A was involved in the occurrence and development of PC (Liu et al., 2012). Besides, in PC cells, TMEM16A expression was inversely correlated with TNF-α expression. TMEM16A silencing could up-regulate the expression of TNF-α, thus promoting the TNF-α signal and eventually leading to the induction of apoptosis and inhibiting the growth of tumor (Song et al., 2018). These findings positioned TMEM16A as an attractive target for anti-cancer therapy.

### Gastric Cancer

GC is a globally important disease. With over 1 million estimated new cases annually, GC is the fifth most diagnosed malignancy worldwide. Due to its frequently advanced stage at diagnosis, mortality from GC is high, making it the third most common cause of cancer-related deaths, with 784 000 deaths globally in 2018 (Bray et al., 2018). Although recent treatments have improved markedly, invasion and metastasis are still the main causes of recurrence and death in GC, which has greatly hampered the effectiveness of treatment (Macdonald, 2006; Yilmaz and Christofori, 2010). However, the understanding of the molecular mechanisms involved in invasion and metastasis of GC is still limited. Yang et al. observed that the positive rate of TMEM16A in 72 human GC tissues with different differentiation levels was 97.22% (70/72). Fang Liu et al. analyzed 367 TMEM16A overexpressed cells (AGS cells and bgc-823 cells) in GC specimens by FISH. It was demonstrated that the overexpression of TMEM16A cells accounted for 69.2% (254/367), and the amplification of TMEM16A was only 7.4% (27/367). note, knockdown of TMEM16A didn’t impact the GC cells proliferation but increased the time required for growth. Subsequently, researchers observed that its knockdown severely inhibited the transfer and invasion of the cells. Another study investigated that there was a negative correlation of the expression of TMEM16A to E-cadherin in patients struggling from GC (Yang et al., 2012). These findings were confirmed by using Western blotting analysis in a subsequently manner, and the protein level of E-cadherin was elevated after the knockdown. Furthermore, the results of clinical studies shown that the amplification and overexpression of TMEM16A showed a positive correlation with tumor node metastasis (TNM) staging and lymph node metastasis. Importantly, TMEM16A knockdown up-regulated E-cadherin by damaging TGF-β secretion to prevent GC cell migration and invasion (Liu et al., 2015). These results suggested that TMEM16A can be utilized as a molecular diagnostic basis and potential therapeutic target for GC.

### Colorectal Cancer

Colorectal cancer (CRC) is the third most common cancer worldwide and one of the leading causes of cancer death (Cheng et al., 2018; Doubeni et al., 2018). CRC’s genes are unstable, and its progress can best reflect the multi-stage transformation of tumorigenesis, including mutation activation of oncogenes and inactivation of tumor suppressor genes (Bray et al., 2018; Zhao et al., 2015). Sui Y. et al. measured TMEM16A expression level in high-metastatic-potential SW620, HCT116 and LS174T cells by using RT-qPCR, Western blotting and immunofluorescence labeling. They demonstrated that the expression of TMEM16A was upregulated in CRC cell lines. Functional assays further demonstrated that TMEM16A-shRNA inhibited the growth, migration and invasion in SW620 cells (Sui et al., 2014). Based on these results, it was found that TMEM16A regulated the proliferation, migration and invasion in CRC cells. Moreover, researchers found that TMEM16A knockdown increased the number of CRC cell at G0/G1 phase and decreased the number of CRC cell at G2/M phase. Hence, the study verified that TMEM16A knockdown blocked cell-cycle progression in SW620 cells, and thus inhibited the growth of TMEM16A shRNA cells (Sui et al., 2014). In conclusion, TMEM16A can promote the growth of CRC cells and can be used as a potential diagnostic marker and therapeutic target.

### Gastrointestinal Stromal Tumor

GIST is a mesenchymal tumor based on the carcinogenic mutation of the stem cell factor receptor (KIT) or the platelet-derived growth factor receptor alpha (PDGFRA) gene (Hirota et al., 1998; Heinrich et al., 2003), which can be treated with a tyrosine kinase inhibitor (TKI), such as imatinib (Simon et al., 2013; Espinosa et al., 2008). In the past, the diagnosis of GIST relied heavily on immunohistochemistry to detect the expression of KIT/CD117 protein. However, in 9 to 15% of GIST, KIT expression does not exist, which may complicate the diagnosis of GIST in patients with beneficial receptor tyrosine kinase inhibitors (Espinosa et al., 2008). In 425 cases of GIST, the positive rate of DOG1.1 (TMEM16A monoclonal antibody) was 87% (370/425) and the reactivity of KIT was 74% (317/428). Furthermore, the high expression of TMEM16A in stromal tumors is more extensive (Espinosa et al., 2008). In the study, only 9% of PDGFRA mutations in GIST (3/32) were positive for KIT, whereas DOG1.1 was positive in most (23/29; 78%) (Espinosa et al., 2008). Based on these data, DOG1 can be used as a tumor-specific target in GIST identification (Simon et al., 2013). As a result, Lee Ch et al. noted that the diagnostic biomarker group helps pathologists and clinicians identify patients who may benefit from targeted therapy when TMEM16A was used in combination with KIT (Lee et al., 2010). With the support of a large number of studies, TMEM16A was found overexpressed in almost all GIST, and that has no significant impact on cell viability and proliferation (Espinosa et al., 2008; West et al., 2004). Interestingly, TMEM16A has pro-apoptotic effects on early apoptotic GIST cells (Berglund et al., 2014). It is noteworthy that TMEM16A knockdown resulted in 96% inhibition of chloride ion outflow in GIST-T1 and 90% inhibition in GIST882 by whole-cell patch-clamp experiments, indicating that TMEM16A is a key regulator for GIST cells to balance chloride balance (Simon et al., 2013). Simon S. et al. found that the inhibition of TMEM16A did not significantly alter the proliferation of GIST-T1 and GIST882 cells by BrdU incorporation assay (Simon et al., 2013). Generally, TMEM16A was highly expressed in GIST cells and had little effect on cell proliferation and survival, but had effect on early apoptosis of advanced GIST cells (Simon et al., 2013). Furthermore, after TMEM16A blockade, the expression profiles of explants showed strong upregulation of IGFBP5 (a potent antiangiogenic factor associated with tumor suppression). The result identified the oncogenic activity of TMEM16A in GIST and involved the regulation of IGF/IGFR signaling in the tumor microenvironment via antiangiogenic factor IGFBP5. Recently, Frobom R et al. demonstrated for the first time that inhibition of TMEM16A expression in vitro can inhibit GIST (Frobom et al., 2019). But at present, the effect of TMEM16A on the metastasis and migration of GIST cells is unclear, and further studies are needed. The wide expression of TMEM16A in GIST indicated that TMEM16A plays an important role in the development of cancer and can be used as a new target for the treatment of GIST.

### Hepatocellular Carcinoma

HCC is a kind of primary liver cancer with high invasion and high mortality. It is one of the most common malignancies in the world, especially in Asia, Africa and southern Europe. About 750,000 people die of liver cancer every year in the world, with China accounting for 51 percent (Yu et al., 2016). Finding an efficient treatment for HCC has become a common goal for researchers around the world. Deng L. et al. found that the expression level of TMEM16A was high in HCC tissues (Deng et al., 2016). Meanwhile, researchers injected SMMC-7721 cells transfected TMEM16A shRNA and NC-shRNA respectively into nude mice. After six weeks, the number of tumors in the TMEM16A and shRNA groups decreased significantly. In addition, the result showed that the G0/G1 phase of TMEM16A siRNA transfected SMMC-7721 cells were increased significantly, but there was no significant change in total apoptosis (Deng et al., 2016). Therefore, researchers believed that high expression of TMEM16A in HCC induced cell cycle arrest, but does not affected apoptosis. It was confirmed that TMEM16A overexpression was used as proto-oncogene to induce tumor growth. Moreover, MTT and invasion assays results showed that knockdown of TMEM16A could significantly inhibited the proliferation of SMMC-7721 cells and decreased SMMC-7721 cells migration and invasion (Deng et al., 2016). Consequently, TMEM16A may be a new biomarker for HCC treatment. Therefore, the development of small molecule inhibitors of TMEM16A may be extremely helpful for the treatment of HCC.

In addition, previous studies have found that the expression level of TMEM16A was increased with the increase of glioma grading (Wang et al., 2017). Additionally, abnormal expression of TMEM16A was found in human lung adenocarcinoma (Jia et al., 2015), ovarian cancer(Liu et al., 2019), acinar cell carcinoma (Chenevert et al., 2012)and pancreatic ductal carcinoma (Sauter et al., 2015; Mazzone et al., 2012). Moreover, TMEM16A can be combined with the traditional periodic acid-Schiff reaction after diastase treatment to distinguish actinic cell carcinomas from other morphologic mimics, particularly mammary analog secretory carcinomas (Chenevert et al., 2012). Increasing evidence indicated that TMEM16A is also considered as a good target and potential biomarker for the treatment of gliomas (Liu et al., 2014), PDAC (Sauter et al., 2015) and LC (Jia et al., 2015). The expression of TMEM16A is significantly different in cancer tissues and normal tissues, which becomes an important clue for finding suitable tumor biomarkers. This effort creates the necessary conditions to improve survival due to untimely diagnosis, metastasis, and recurrence are undoubtedly the biggest problems in the cancer field. However, the role of TMEM16A in cancer is controversial, so the effect of TMEM16A in tumorigenesis and development needs to be clarified. In addition, more aspects of clinical application and larger clinical trials are essential. Finally, the use of TMEM16A as a biomarker for cancer has been gradually observed, which provides a new basis for the early diagnosis or treatment of cancer in the future.

## The Regulatory Mechanism of TMEM16A in Cancer

**FIGURE 3:**
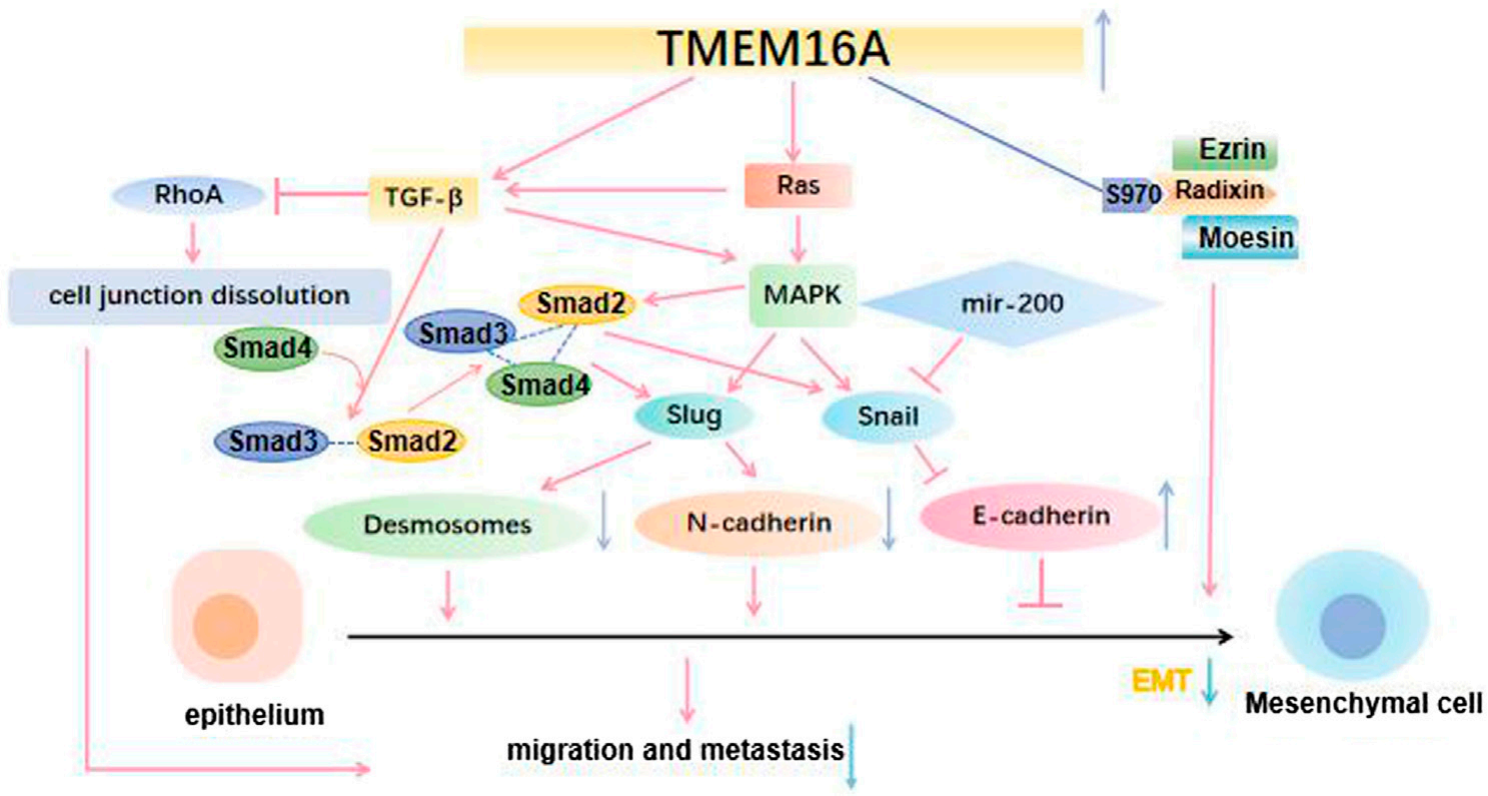
TMEM16A regulates cancer cell proliferation. 3.1 TMEM16A interacts with EGFR on the cell membrane, stimulates the Ras-Raf-MEK signaling pathway and acts on receptors in the nucleus such as STST3, AP-1; 3.2 TMEM16A activates the CAMK signaling pathway, which in turn activates the Akt signaling pathway. Both pathways promote the expression of CCND1 and ultimately the proliferation of cancer cells.

**FIGURE 4:**
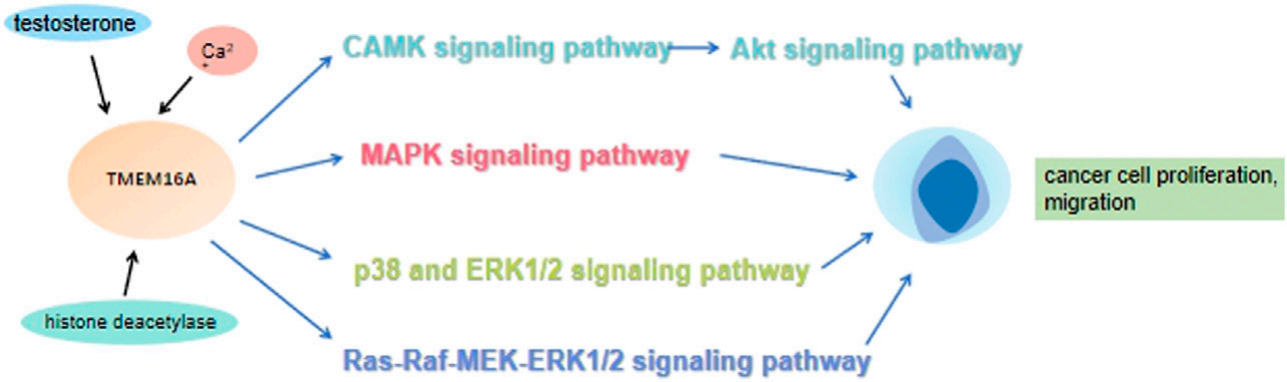
Under the action of testosterone, histone deacetylase and calcium ions, TMEM16A promotes the proliferation and migration of cancer cells by regulating CAMK-Akt, MAPK, p38, ERK1/2, Ras-Raf-MEK-ERK1/2 and other signaling pathways.

It is well known that improving patient survival requires a better understanding of tumorigenesis and metastasis, as well as effective biomarkers for determining cancer occurrence and metastasis. In addition, If these aggressive tumors can be detected at an early stage and treated in a targeted manner, the mortality rate could be significantly reduced. Increasing studies have shown that many signaling pathways are involved in the occurrence and development of cancer. Of note, the MAPK signaling pathway can promote cancer cells proliferation via elevating the cyclin D1 (CCND1) expression (Figure 3). The activation of MAPK signaling pathway also can promote the angiogenesis of vascular endothelial cells. Previous studies have shown that new angiogenesis can provide more nutrition for tumors, accelerate tumor growth and promote the proliferation of cancer cells. Mechanically, MAPK signaling pathway transmits the upstream extracellular growth factor signal to a variety of downstream effectors located in the nucleus, regulating cell proliferation, differentiation, survival and apoptosis (Dhillon et al., 2007). The conformation change of RAS could activate MAPK signaling pathway. Under the stimulation of upstream receptors, the RAS (inactive) bound to guanosine diphosphate (GDP) is converted to RAS (active) that binds to guanosine triphosphate (GTP), resulting in the synthesis and activation of RAF. Subsequently, activated RAF phosphorylates MEK, which directly leads to ERK phosphorylation. Finally, activated ERK phosphorylates a variety of substrates, including kinases, transcription factors and so on (Liu et al., 2018). Numerous research results showed that TMEM16A participates in the progress of various diseases by regulating EGFR/STAT3 signaling pathways. TMEM16A appears to activate distinct signaling pathways in different cancers (Wang et al., 2019). For example, TMEM16A activates the NF-κB signaling pathway in glioma (Liu et al., 2014), the Ras-Raf-MEK-ERK1/2 signaling pathway in HNSCC (Duvvuri et al., 2012) and the p38 and ERK1/2 signaling pathways in hepatoma (Deng et al., 2016). Lian H et al. theorized that TMEM16A increased podocyte apoptotic rate in diabetic nephropathy mice through inducing P38/JNK signaling pathway activation, which exacerbates renal injury (Lian et al., 2017). Furthermore, Li R. S. et al. suggested that TMEM16A contributes to angiotensin II-induced cerebral vasoconstriction by the RhoA/ROCK signaling pathway (Li R. S. et al., 2016). In previous studies, it was found that the up-regulated TMEM16A could promote cancer by activating a signaling pathway. As a CaCC, membrane voltage (vm), intracellular calcium concentration and external permeable anions promoted the TMEM16A activation (Ji et al., 2019). A large number of experiments have also confirmed that Ca^2+^ seems to play a greater role in activating phospholipase-C (PLC) generates IP3, whose receptor IP3R mediates the release of internal store Ca^2+^, and finally activates the EGF-induced Ca^2+^ reaction. TMEM16A interacts with IP3R and is activated by the release of Ca^2+^ mediated by IP3R (Jin et al., 2013). In addition, TMEM16A can also be activated by Ca^2+^ flowing into extracellular media (Sun Y. et al., 2015). These results suggested that TMEM16A may play a role in regulating EGF-induced Ca^2+^ signaling pathway, and may be particularly related to the engagement of TMEM16A with EGFR (Crottes et al., 2019). Mechanistically, TMEM16A interacts with EGFR on the cell membrane and activates EGFR. The activated EGFR binds to the SHC-Grb2-SOS complex and further stimulates the RAS-Raf-MEK-ERK signaling pathway, acting on receptors in the nucleus, such as STAT3, AP-1 (Kulkarni et al., 2017). CAMKII is a multifunctional Ca^2+^ and cam dependent serine/threonine protein kinase, which is linked with cell proliferation (Gangopadhyay et al., 2003). Thus, TMEM16A also can activate the CAMK signaling pathway directly, which in turn activates the Akt signaling pathway. Finally, together with the MAPK signaling pathway, it promotes the expression of CCND1, and then the proliferation of cancer cells was promoted (Sui et al., 2014) (Figure 4).

Generally, cancer cells with TMEM16A overexpression due to 11q13 gene amplification exhibit higher proliferative capacity than those without 11q13 gene amplification, since the 11q13 amplicon contains a variety of other genes whose expression may contribute to the cellular changes that drive 11q13 amplified cancer cells to a proliferative state (Huang et al., 2006). In addition to amplification of the amplicon in which it resides, TMEM16A expression and function are epigenetically regulated, including promoter methylation and microRNA. On the one hand, researchers found that TMEM16A promoter hypermethylation promoted metastasis but inhibited proliferation of TMEM16A overexpressing cancer cells, with opposite effects when the promoter was hypomethylated (Shiwarski et al., 2014; Dixit et al., 2015). On the other hand, researchers found that TMEM16A was the direct target of miR-132 and TMEM16A overexpression was inversely associated with downregulation of miR-132 in human CRC (Mokutani et al., 2016). Besides, miR-381 in human GC had a similar regulatory effect on TMEM16A as well (Cao et al., 2017). In addition to the intrinsic mechanisms described above, TMEM16A also achieves specificity of its effects in different cancers through the heterogeneity of downstream signaling pathways, such as the p38 and ERK1/2 signaling pathway in hepatoma (Deng et al., 2016), the Ras-Raf-MEK-ERK1/2 signaling pathway in HNSCC and bladder cancer (Duvvuri et al., 2012), and the NF-κB signaling pathway in glioma (Liu et al., 2014). In conclusion, multiple mechanisms including 11q13 gene amplification, epigenetic regulation and signaling pathways determine the cell specificity of TMEM16A expression and function.

## Potential Clinical Application of TMEM16A in Cancer.

In order to achieve early and effective diagnosis, a clear tumor biomarker pair is essential when monitoring tumor molecular differences. Cancer’s survival rate is low bacause its best surgical opportunity is not easy to grasp, because it is difficult to detect at an early stage. In the clinic, prognostic markers can be used to predict clinical outcomes in poorly treated cancer patients. On the other hand, Current research has explored the feasibility of TMEM16 family expression profiles in predicting cancer or distinguishing cancer subtypes. However, However, it cannot accurately predict tumor progression due to its uncertain molecular biological function. TMEM16A is involved in many biological functions of cancer cells (Frobom et al., 2019; Ji et al., 2019). An ideal biomarker should have several typical characteristics. For example, the Cancer Genome Atlas (TCGA) data shown that TMEM16A was high expression in about 30% of HNSCC (Gao et al., 2013; Cerami et al., 2012). After analyzing the maximum values of TMEM16A H in two clinical subgroups, the results revealed that it was consistent with the TCGA (n = 7/27, 25.9%). The proportion of TMEM16A overexpression (n = 10/14, 71%) was significantly increased (Godse et al., 2017). More importantly, the abnormal expression TMEM16A was significantly linked with poor overall survival of patients with GC (Liu et al., 2015), esophageal cancer (Shang et al., 2016), and CRC (Mokutani et al., 2016). For example, the related research reported that higher expression of TMEM16A was previously shown to be associated with decreased survival (Finegersh et al., 2017). Upregulation of TMEM16A expression was linked with low overall survival and high morbidity of BC patients (Britschgi et al., 2013). In addition, studies showed that TMEM16A gene amplification and protein overexpression affect the clinical outcomes of HNSCC patients (Ruiz et al., 2012), especially HPV-negative HNSCC patients (Dixit et al., 2015). More importantly, a meta-analysis of the microarray data set revealed that TMEM16A is a poor prognostic marker for HNSCC (Reddy et al., 2016). The above studies have found that the overexpression of TMEM16A is related to the poor prognosis of cancer patients, so TMEM16A can serve as a biomarker for the clinical prognosis of cancer patients.

Recently, researchers showed that good prognosis could be detected via abnormal expression of TMEM16A in BC patients who are PR-positive or HER2-negative after tamoxifen treatment, especially in those patients along with the silence of Ki67 (Wu et al., 2017; Wu et al., 2015). Studies demonstrated that combined with clinically relevant markers such as ER, PR, HER2 and Ki67, the expression of TMEM16A may help predict the clinical prognosis of BC patients. In addition. Kulkarni et al. found that its inhibition improves the response of HNSCC cells to EGFR / HER2 targeted therapies (Kulkarni et al., 2017). Moreover, Li et al. reported that TMEM16A expression was elevated in circulating tumor cells (CTCs) in patients with relapsed GIST than in patients with relapse-free GIST and related to disease-free survival (Li Q. et al., 2016). In short, the expression of TMEM16A can predict the efficacy of EGFR/HER2 inhibitors in BC and HNSCC patients and monitor the recurrence situation, and can be used as a biomarker to predict the efficacy of imatinib in treating GIST patients. In addition, Shi Z. Z. et al. found that the mRNA expression level of TMEM16A in ESCC was significantly higher than that in adjacent tissues (Ji et al., 2019). Similarly, IHC results further indicated that TMEM16A is overexpressed in ESCC at 25% (n = 88) and the higher expression of TMEM16A is significantly associated with lymph node metastasis and advanced tumor staging. The expression of TMEM16A was confirmed to be related to the progression of precancerous lesions in 148 cases of intraepithelial lesions, and three-quarters of patients with high TMEM16A expression developed ESCC (34 cases) within 4-9 years after initial diagnosis.

Furthermore, Duvvuri et al. with the support of the tumor amine database to determine the expression of TMEM16A in normal and malignant tissues of various tumors (Duvvuri et al., 2012). They found that TMEM16A overexpression in 80% of head and neck squamous cell carcinoma (HNSCC), which correlated with decreased overall survival in patients with HNSCC. TMEM16A overexpression significantly promoted anchorage independent growth in vitro, and loss of TMEM16A resulted in inhibition of tumor growth both in vitro and in vivo. (Duvvuri et al., 2012). More importantly, they determined that overexpression of TMEM16A is related to clinical outcomes in HNSCC patients. In HNSCC tumors, about 85% of TMEM16A expression was found. Kaplan-Meier survival analysis showed that tumor patients with high expression of TMEM16A had lower overall survival rates (P<0.04, log-rank test). Interestingly, poor overall survival was induced by overexpression of TMEM16A in a univariable Cox proportional hazards model (HR ¼ 3.04; 95% CI, 0.97–9.46) and in a multivariable Cox proportional hazards model adjusting for age, sex, and nodal stage (HR ¼ 2.8; 95% CI, 0.86–9.16) (Duvvuri et al., 2012).

The biggest difficulty of cancer treatment lies in the late diagnosis time, easy to relapse and metastasis. Therefore, it is important for the early diagnostic rate to search for the ideal biomarker of cancers. The above findings indicated that TMEM16A might be acted as a potential biomarker for the PC, LSCC and GC diagnosis. However, it should not be used solely, but interpreted in conjunction with diagnostic imaging, clinical history, and physical examination. For example, current test results for CA125 in the clinic need to be combined with ultrasound findings to be used for ovarian cancer screening in women older than 50 years. In general, decreasing TMEM16A levels following initiation of therapy correlates with tumor regression and conversely elevation of TMEM16A should lead to a high index of suspicion for progression and/or recurrent tumor. However, although TMEM16A can be elevated in the setting of cancer, some patients may not show its elevation, and, on the other hand, benign conditions can also cause false positive elevation of TMEM16A. Therefore, we are supposed to be aware of the limitation of TMEM16A as a tumor marker. Of course, combined application with other tumor markers can offset this limitation to some extent. For example, the combined detection of AFP and AFU is often used in clinic at present to improve the diagnostic rate of HCC. On the technical level, we can make TMEM16A detection kits for clinical use. At present, TMEM2, TMEM14B, TMEM27, TMEM59, TMEM119, TMEM173 and many more TMEM family detection kits have been developed and put into use. Most of them employ enzyme-linked immunosorbent as the detection principle with high sensitivity and specificity. However, from what we have retrieved and learned, except for TMEM27, other TMEM protein detection kits are for research use only and are not available for clinical use. This is largely due to the lack of TMEM protein antibodies of human origin, and the significant differences in TMEM protein between species make cross reactivity unlikely. Therefore, if in the future we want to develop a detection kit for TMEM16A, it is first necessary to obtain human antibodies to TMEM16A. Furthermore, for the detection of TMEM16A with a large sample size, tissue microarray is also a good choice.

## Future Expectations

Notably, many studies have demonstrated the material role of TMEM16A in the development of cancer. Therefore, more and more cancer researchers will focus on this protein. Recent studies have displayed that TMEM16A participates in the development of tumors, and may be acted as a target for the monitoring of cancer detection and treatment marker. Of note, epigenetics is related to the occurrence and development of cancer (Lee et al., 2018). Therefore, as the main content of epigenetics, DNA methylation and microRNA are the directions for many researchers. It is well documented that DNA methylation is a common post-replication modification in prokaryotic and eukaryotic genomes, involving a variety of essential physiological processes in the body, such as regulating gene expression, maintaining chromosome integrity, and gene imprinting (He et al., 2018; Kidder et al., 2017; Goovaerts et al., 2018). Shiwarski D. J. et al. found that the TMEM16A expression was declined in metastatic tissue compared with primary cancer tissue. Subsequently, the methylation of the TMEM16A promoter could regulate the protein level of TMEM16A in cancer cells (Shiwarski et al., 2014). In addition, Dixit R. et al. also found that the expression level of TMEM16A in HPV-positive HNSCC is lower than that of HPV-negative HNSCC due to the higher degree of promoter methylation (Dixit et al., 2015). Based on these results, we speculate whether methylase can change the methylation level of TMEM16A transcription promoter, thereby regulating its expression level and thus regulating tumor progression. On the other side of epigenetics, the expression of TMEM16A in cancer cells is associated with microRNA. Cao Q. et al. found that miR-381 can directly target TMEM16A, inhibit TGF-β signaling pathway and downregulate EMT, thereby inhibiting the proliferation, metastasis and migration of GC cells (Cao et al., 2017). Similarly, Sonneville F. et al. found that miR-9 was negatively correlated with the expression of TMEM16A in the bronchial epithelial cells of cystic fibrosis (CF) (Sonneville et al., 2017). The result also showed that TMEM16A is an immediate target of miR-9 in the bronchial epithelial cells of CF. MiRNA plays an important role in the physiological and pathological processes such as cell differentiation, proliferation, apoptosis and angiogenesis (Lin and Gregory, 2015; Jovanovic and Hengartner, 2006). Most miRNAs regulate target miRNAs by binding to the 3′-UTR of the target gene after translation, and miRNAs are therefore degraded or inhibited after translation (Zhou et al., 2017). For example , various miRNAs (including miR-21 , miR-34a , miR-155 , and miR-29a ) have been demonstrated to be linked with tumor progression and overall survival in PC patients (Sun X. J. et al., 2015). MiR-449a suppressed the growth and metastasis of endometrial cancer by directly targeting the NDRG1 gene (Wu et al., 2019) and miR-26a promoted invasion/metastasis by inhibiting PTEN and inhibited cell proliferation by repressing EZH2 in HCC (Zhao et al., 2019). Taken together, we have reasons to believe that the corresponding miRNA in cancer cells is the expected direction of cancer treatment by targeting TMEM16A in the future.

Notably, gene editing, a hot topic in current research, may also provide an effective means for cancer treatment. Takao A. et al. shown that the lack of PTEN can mobilize a variety of cancer cell survival and host immunity. Obtaining a PTEN gene knockout cell line (ΔPTEN) from a mouse PC cell line suggests that gene editing systems such as CRISPR or CRISPR can be used to evade important genes/Cas9, clustered regularly spaced short palindromic repeats/CRISPR-related protein 9 (Takao et al., 2018). Crispr-cas9 drive tet1 and dnmt3a are delivered to specific DNA sequences and are now the latest mechanism for studying DNA methylation (Liu et al., 2016). Finegersh A et al. suggested that the level of ANO1 is associated with CpG’s at three CpG islands annotated by the UCSC genome browser, and was greater effected by the positively correlated CpG. Further research on CRISPR-Cas9 mediated CpG transfer from TET1 and DNMT3A to TMEM16A may establish a mechanism connection between DNA methylation and expression (Finegersh et al., 2017). Considering that TMEM16A could regulate the progression and development of cancer, if TMEM16A is used as the potential target gene for gene editing in the future, there may be a new prospect for cancer therapy.

At present, TMEM16A has been widely used in the prognostic study of BC and HNSCC, and its abnormal expression is closely linked with the prognosis of tumor. But for TMEM16A, we still have many questions. For example, 1) We know that there are many factors that can promote the high expression of TMEM16A. For example, transcriptional stimulation of the IL4/IL13-Jack-STAT3-STAT6 axis, activation of steroid hormones such as testosterone, histone deacetylase (HDCA), promoter hypomethylation, and downregulation of inhibitory miRNA have been shown to increase the expression of TMEM16A (Wanitchakool et al., 2014; Bill and Alex Gaither, 2017; Qu et al., 2014; Fujimoto et al., 2018). Is it possible to prevent or treat cancer through this stimulus? 2) Is the high expression of TMEM16A related to the stimulation of its gene enhancer? Can we proceed to treat cancer? 3) As a protein on cell membrane, can TMEM16A be used as a target for new drug discovery? Although the abnormal expression of TMEM16A in cancer for clinical detection and treatment of cancer to bring hope, but its role in different cancers is not the same, what is the cause of this difference is unknown; the future more research is needed to uncover its mysterious.

## Conclusion

Increasing evidence shows that TMEM16A can regulate a variety of cellular processes, physiological responses and human diseases, especially in cancer (Ruppersburg and Hartzell, 2014; Deba and Bessac, 2015). Multiple studies demonstrated that uncontrolled cell cycle progression, survival, proliferation, growth, migration, invasion, EMT and metastasis were regulated by TMEM16A (Wang et al., 2017; Shiwarski et al., 2014). The aberrant expression of TMEM16A in malignant tumors suggested the opportunity for developing it as a clinical biomarker for prevention and early detection of cancer. In addition, it is also involved in regulating cancer cell signaling pathways. In BC, TMEM16A regulated cell proliferation via modulating EGFR and CAMK signaling pathways (Wu et al., 2017). In HNSCC, TMEM16A is significantly increased in primary tumors. The proliferation of cancer cells was promoted. However, the expression of TMEM16A is reduced in lymph node metastasis, and increased migration and invasion (Shiwarski et al., 2014). In addition, TMEM16A also plays a considerable role in LC, PC, glioma and other malignant tumors (Liu et al., 2015; Liu et al., 2014; Guo et al., 2019; Jun et al., 2017). In summary, it can regulate the life course of tumors, and the abnormal expression of TMEM16A in malignant tumors provides the possibility to prevent tumors and find clinical biomarkers for early diagnosis. Research on larger samples and more precise mechanisms will be however the main trend and focus. In this context, TMEM16A is expected to eventually achieve clinical application.
